# Global MicroRNA Profiling Uncovers miR-206 as a Negative Regulator of Hematopoietic Commitment in Human Pluripotent Stem Cells

**DOI:** 10.3390/ijms20071737

**Published:** 2019-04-08

**Authors:** Stéphane Flamant, Jean-Claude Chomel, Christophe Desterke, Olivier Féraud, Emilie Gobbo, Maria-Teresa Mitjavila-Garcia, Adlen Foudi, Frank Griscelli, Ali G. Turhan, Annelise Bennaceur-Griscelli

**Affiliations:** 1 INSERM UMR-S935, F-94807 Villejuif, France; stephane.flamant@irsn.fr (S.F.); jean-claude.chomel@chu-poitiers.fr (J.-C.C.); christophe.desterke@inserm.fr (C.D.); olivier.feraud@inserm.fr (O.F.); maite.mitjavila@inserm.fr (M.-T.M.-G.); frank.griscelli@gmail.com (F.G.); turviv33@gmail.com (A.G.T.); 2 Institut de Radioprotection et de Sûreté Nucléaire (IRSN), F-92262 Fontenay-aux-Roses, France; 3 Service de Cancérologie Biologique, CHU de Poitiers, F-86021 Poitiers, France; 4 Faculté de Médecine, Université Paris Sud, Paris Saclay, F-94270 Le Kremlin-Bicêtre, France; 5 INGESTEM-ESTeam Paris Sud, F-94800 Villejuif, France; gobbo.emilie@wanadoo.fr; 6 ATIP-Avenir, INSERM UMR-S 935, Université Paris Sud, F-94800 Villejuif, France; adlen.foudi@inserm.fr; 7 Faculté des Sciences Pharmaceutiques et Biologiques, Université Paris Descartes, Sorbonne Paris Cité, F-75000 Paris, France; 8 Service d’Onco-Hématologie, AP-HP Hôpitaux Universitaires Paris Sud, Hôpital Paul Brousse, F-94804 Villejuif, France; 9 Service d’Hématologie, AP-HP Hôpitaux Universitaires Paris Sud, Hôpital Bicêtre, F-94270 Le Kremlin-Bicêtre, France

**Keywords:** Human pluripotent stem cells, embryonic stem cells, induced pluripotent stem cells, hematopoietic potential, microRNAs, miR-206

## Abstract

Although human pluripotent stem cells (hPSCs) can theoretically differentiate into any cell type, their ability to produce hematopoietic cells is highly variable from one cell line to another. The underlying mechanisms of this heterogeneity are not clearly understood. Here, using a whole miRNome analysis approach in hPSCs, we discovered that their hematopoietic competency was associated with the expression of several miRNAs and conversely correlated to that of miR-206 specifically. Lentiviral-based miR-206 ectopic expression in H1 hematopoietic competent embryonic stem (ES) cells markedly impaired their differentiation toward the blood lineage. Integrative bioinformatics identified a potential miR-206 target gene network which included hematopoietic master regulators RUNX1 and TAL1. This work sheds light on the critical role of miR-206 in the generation of blood cells off hPSCs. Our results pave the way for future genetic manipulation of hPSCs aimed at increasing their blood regenerative potential and designing better protocols for the generation of bona fide hPSC-derived hematopoietic stem cells.

## 1. Introduction

Although pluripotent stem cells (PSCs) can differentiate into practically all mature cells of the body, it is now well-established that they display some variations in their differentiation potential toward specific cell lineages. This phenomenon depends on derivation, culture and differentiation conditions, as well as genotype, transgene reactivation, or the epigenetic signature of reprogrammed cells [1,2,3]. For example, a study involving 17 independent PSC lines derived at the Harvard University revealed marked differences in their propensity to differentiate into specific cell lineages, during both spontaneous and guided differentiation assays [4].

Research aimed at differentiating human embryonic or induced pluripotent stem cells (hESCs or hiPSCs) toward mature hematopoietic cells for disease modeling and therapeutic purposes is actively ongoing. However, several issues deserve consideration such as the development of a definitive human hematopoiesis by in vitro differentiation of hPSCs or the optimization of protocols to generate clinical-grade mature blood cells [5,6]. Recently, the identification of factors playing a critical role in the production of hematopoietic engraftable stem cells and progenitors from human PSCs yielded substantial improvements in attempts to obtain mature functional peripheral blood cells [7].

Nonetheless, some methods to study the early steps of hematopoiesis from human ES and iPS cells have been applied, but with a high degree of variability according to the protocols being used. Classically, hPSCs are differentiated into embryoid bodies (EB) in the presence of hematopoietic cytokines, supplemented or not with mesoderm-inducing factors such as BMP4 or ACTIVIN. Under these conditions, CD31+/CD45– hemogenic progenitors emerge within ten days of culture, while CD34+/CD45+ hematopoietic progenitors are detected in 12- to 16-day old EBs [8,9]. A study applying this method to a panel of 13 hESC and one hiPS cell lines demonstrated striking differences in their ability to differentiate toward blood cell lineage, with some PSCs generating robust hematopoiesis while others showed poor hematopoietic potential [10]. Interestingly, this discrepancy was not associated with the expression of hematopoiesis-related genes but rather linked to genes involved in the NODAL/ACTIVIN signaling pathway, which induces the mesendodermal specification of hPSCs [11].

MicroRNAs (miRNAs) are a class of small non-coding RNA molecules, which post-transcriptionally repress gene expression through partial base pairing with the targeted transcript. Over the last 15 years, miRNAs have been involved in virtually every physiological and developmental cell process, including hematopoiesis [12,13] and maintenance of pluripotency [14]. In addition, the importance of miRNAs in both iPS cell reprogramming and hematopoiesis regulation was recently highlighted [15]. We previously showed that hES and hiPS cell lines of diverse origins displayed significant intrinsic differences in their ability to differentiate toward the hematopoietic lineage, regardless of the reprogramming method or expression of key transcription factors [16].

The purpose of this study was to explore the molecular basis of human pluripotent stem cells’ hematopoietic differentiation potential. We explored the putative role of microRNAs as biomarkers to identify hPSCs that are more amenable for hematopoietic cell differentiation using a panel of 11 hiPSCs and 4 hES cell lines. First, hPSC hematopoietic potential was explored using clonogenic assays, flow cytometry and transcriptome analysis focused on genes involved in hematopoiesis, germ layer differentiation, and the NODAL/ACTIVIN signaling pathway. Furthermore, we analyzed the expression of blood-related miRNAs during EB differentiation on a subgroup of hPSCs. Overall miRNome analysis of the entire panel of 15 hPSCs was then carried out at the pluripotent undifferentiated stage, revealing a clear association between the expression of specific miRNAs and the hematopoietic potential of hPSCs. Amongst these, miR-206 was significantly and consistently repressed in hematopoietic-competent hPSCs. Through in vitro ectopic expression of miR-206 in blood-competent ES cells and *silico* characterization of miR-206 target genes, we have established the critical role of this miRNA in hematopoietic lineage output of hPSCs.

## 2. Results

### 2.1. Overview of the Protocol

Four hESC and 11 hiPSC lines were analyzed in this study (Table 1). Human PSCs were assayed after an average of 33 passages and differentiated into hematopoietic progenitors from EBs, using established hematopoietic permissive culture conditions. Their hematopoietic potential was evaluated by flow cytometry, colony formation, and whole transcriptome analysis in day-16 EBs. Two sub-groups of hPSCs were thereby identified according to their hematopoietic competence.

**Figure 1:**
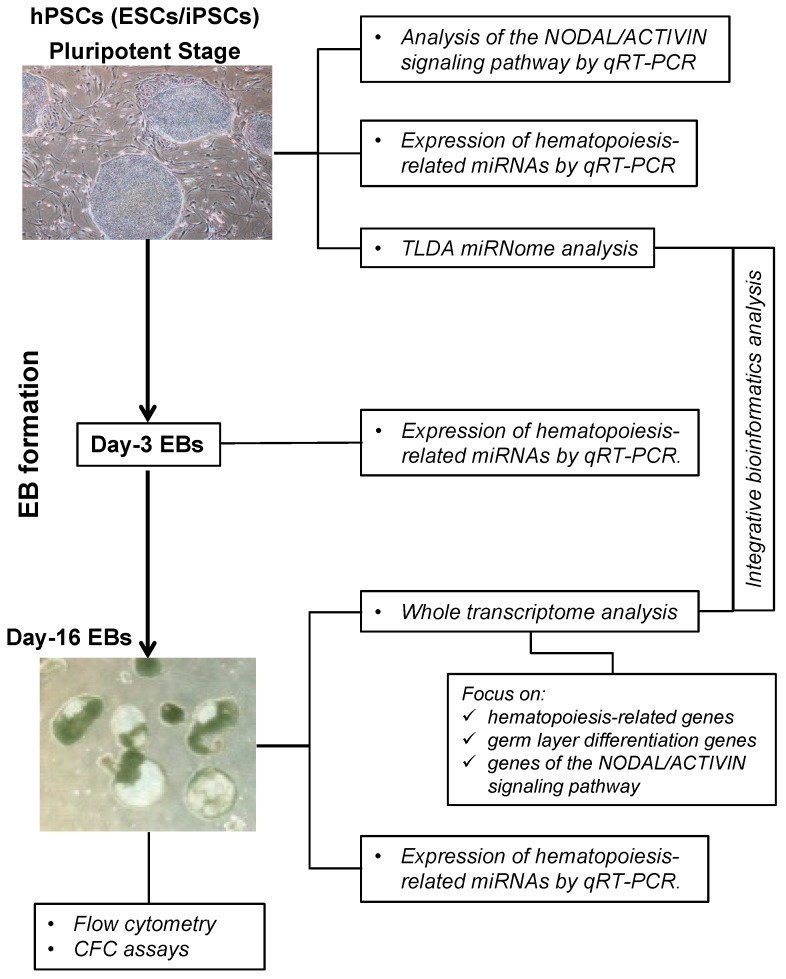
Comprehensive analysis of the hematopoietic potential of human pluripotent stem cells. Human pluripotent stem cells (hPSCs) were differentiated into embryoid bodies (EBs) using appropriate hematopoietic factors. Flow cytometry, colony-forming cell assays and gene expression analysis were performed on day-16 EBs. For miRNA expression kinetic analysis, hPSCs or EBs were collected at day 0, 3, and 16 of culture. The global miRNome analysis was carried out on hPSCs at pluripotent stage. ESCs, embryonic stem cells; iPSCs, induced pluripotent stem cells; TLDA, TaqMan Low Density Array; CFC, colony-forming cell.

Hematopoiesis-related miRNA expression in EBs was followed during the differentiation process by quantitative RT-PCR at day 3 and 16. TLDA (TaqMan low-density array) miRNome analysis was then performed at the pluripotent undifferentiated stage in both subgroups (Figure 1). These data were crossed in an integrative bioinformatic analysis with day-16 EB transcriptomic data. In addition, the expression level of genes involved in NODAL/ACTIVIN pathways was carried out in EBs and hPSCs.

### 2.2. Characterization of the Hematopoietic Potential of hPSC Lines

The hematopoietic potential of human ES and iPS cell lines was determined using colony formation assays and flow cytometry in day-16 EBs (Table 2). Four iPS cell lines (PB6, PB9, PB12.1, and PB14.3) were totally lacking in hematopoietic differentiation ability. Among hPSC lines displaying hematopoietic potential, two sub-groups were further defined based on their colony formation efficiency: A group of poor hematopoietic-competent hPSCs (less than 100 colonies/105 cells, PB4/PB10/H9/CL01), and a group of high hematopoietic-competent hPSCs (more than 120 colonies/105 cells, PB3/PB6.1/PB7/PB13/PB17/SA01/H1). Interestingly, as previously described [16], hPSCs were intrinsically heterogeneous, giving rise to either erythropoiesis, granulopoiesis or all blood cell types.

Day-16 EB cells from 3 hematopoietic-competent hPSC lines (H1, PB6.1, PB7) and two hematopoietic-deficient iPSC lines (PB6, and PB12.1) were analyzed by whole gene expression analysis using microarrays with focus on hematopoiesis-related and germ layer differentiation genes (Table S1). Unsupervised hierarchical clustering segregated hematopoietic-deficient from -competent cells based on hematopoietic lineage markers (Figure S1). Expression levels of several genes encoding either key proteins such as *CD34* or master transcription factors such as *SPI1/PU.1*, *GATA1*, *TAL1*, *RUNX1* were found down-regulated in hematopoietic-deficient iPSC-derived EBs. The same samples were also tested for their capability to differentiate into endoderm, mesoderm or ectoderm (Figure S2). In this context, several genes involved in mesoderm (*KDR/VEGFR2*, *HAND1*, *COL1A1*) or endoderm (*GATA4*, *GATA6*, *FN1*, *FOXA2*, *EPCAM*) differentiation appeared up-regulated in hematopoietic-competent hPSCs. Intriguingly, *SOX2* was previously described to be down-regulated during hematopoietic development, with its expression inversely correlated to the hematopoietic potential of PSCs [17]. However, we found no significant change in *SOX2* expression level between hematopoietic-competent and -deficient hPSC lines in our study.

### 2.3. Gene Expression Analysis of the NODAL/ACTIVIN Signaling Pathway

This pathway belongs to the TGF-beta signaling pathway and is involved in many developmental processes, including hematopoiesis (Figure S3A). The mRNA levels of several genes from the NODAL/ACTIVIN/BMP pathways were evaluated by microarray analysis in day-16 EBs from H1, PB6, PB6.1, PB7, and PB12.1 hPSCs, and by quantitative RT-PCR in all 15 hPSC lines at the pluripotent undifferentiated stage (Table S2 and Figure S3B). None of these genes were differentially altered either in EBs or at the pluripotent stage. Hence, they did not enable us to discriminate hematopoietic-deficient from -competent hPSCs solely based on their expression (Figure S3C,D).

### 2.4. Hematopoiesis-Related miRNA Expression during Hematopoietic Differentiation

**Figure 2:**
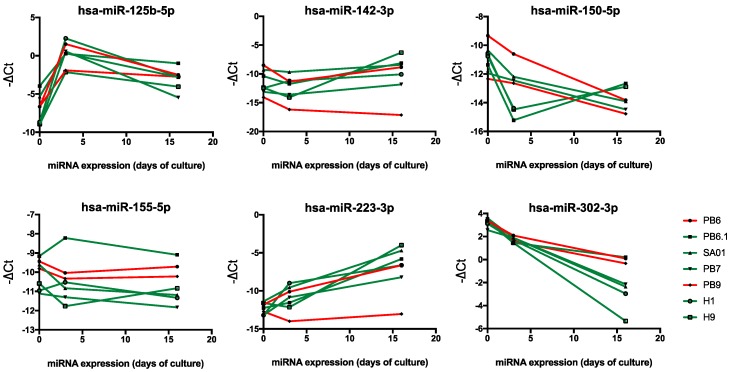
Hematopoiesis-related miRNA expression during EB culture. Five hematopoietic-competent PSCs (PB 6.1, PB7, SA01, H1, H9) and two hematopoietic–deficient ones (PB6, PB9) were analyzed at 0, 3 and 16 days in the course of hematopoietic differentiation (day 0 representing the undifferentiated stage) by qRT-PCR. Graphs represent the expression kinetics of hsa-miR-125b-5p, hsa-miR-142-3p, hsa-miR-150-5p, hsa-miR-155-5p, hsa-miR-223-3p, and the hPSC-specific miR-302-3p, estimated by a –ΔCt calculation (with ΔCt = Ct miRNA – Ct RNU48). Hematopoietic-competent and hematopoietic-deficient PSCs are represented by green and red lines, respectively.

The role of miRNAs has been extensively explored in adult tissues including hematopoietic compartment, with functions in stem cell self-renewal, differentiation and in hematological disorders such as acute myeloid leukemia. Aside from their putative function, the role of miRNAs in early hematopoietic development has yet to be explored. As cell reprogramming and differentiation may be altered by miRNA expression, we have investigated the kinetics of hematopoiesis-related miRNA expression in hESC and hiPSC during hematopoietic commitment (Table S3). The expression kinetics of five miRNAs with recognized role in hematopoiesis (hsa-miR-125b-5p, hsa-miR-142-3p, hsa-miR-150-5p, hsa-miR-155-5p, hsa-miR-223-3p) and those of the PSC-specific hsa-miR-302-3p (used as control) were analyzed in hematopoietic-deficient (PB6, PB9) and -competent hPSCs (PB 6.1, PB7, SA01, H1, H9), at the pluripotent undifferentiated stage (day 0) and in day-3 and day-16 EBs (Figure 2). As expected, miR-302 expression decreased upon hPSC differentiation into EBs.

Interestingly, miR-302 expression level remained elevated in hematopoietic-deficient PB6 and PB9 iPSCs, as compared to most hematopoietic-competent cells. Expression of miR-125b, related to multipotent HSC, was increased early in day-3 EBs and partially reduced in day-16 EBs. Blood-specific miR-223 was largely up-regulated in day-16 EBs, whereas the relative expression of miR-142 appeared to be somewhat stable. Notably, the hematopoietic-deficient PB9 iPSC line displayed a reduced expression level of miR-223 and miR-142 in both day-3 and day-16 EBs. We also noted substantial variations among the PSC lines regarding the expression of miR-155 and miR-150 (Figure 2).

### 2.5. Global microRNA Expression Profiling in Human PSCs

To demonstrate a predictive value of miRNAs as markers of hematopoietic potential, the expression of 754 individual miRNAs was analyzed in our 15 hPSC lines at the pluripotent stage. Clustering gene expression patterns were determined using hierarchical algorithms of StatMiner software applying Euclidean distance and Ward’s linkage method. This unsupervised method did not segregate hPSCs according to their hematopoietic potential (Figure S4). An initial supervised study comparing miRNA expression between ESCs and iPSCs was performed to ensure the homogeneity of our series of hPSCs for further analyses. In such a case, no significant variation in miRNA expression was observed using the LiMMa (Linear models for microarrays) method and Bonferroni correction. This result implies that our panel of 15 hPSCs consisting of 4 ESCs and 11 iPSCs was sufficiently homogeneous to allow reliable miRNA expression analyses.

### 2.6. Human PSCs Hematopoietic Potential and miRNA Expression

**Figure 3:**
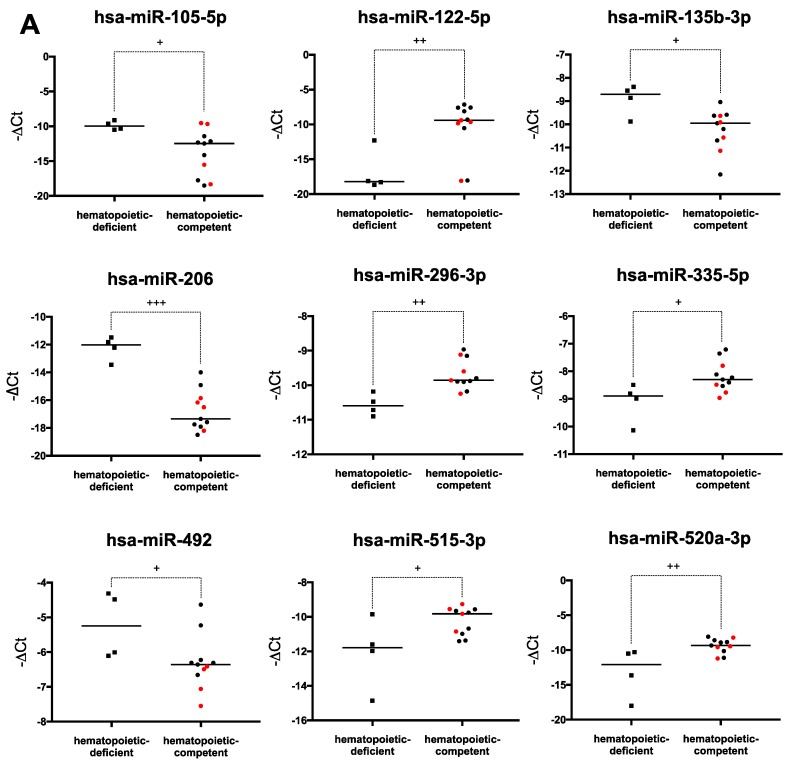

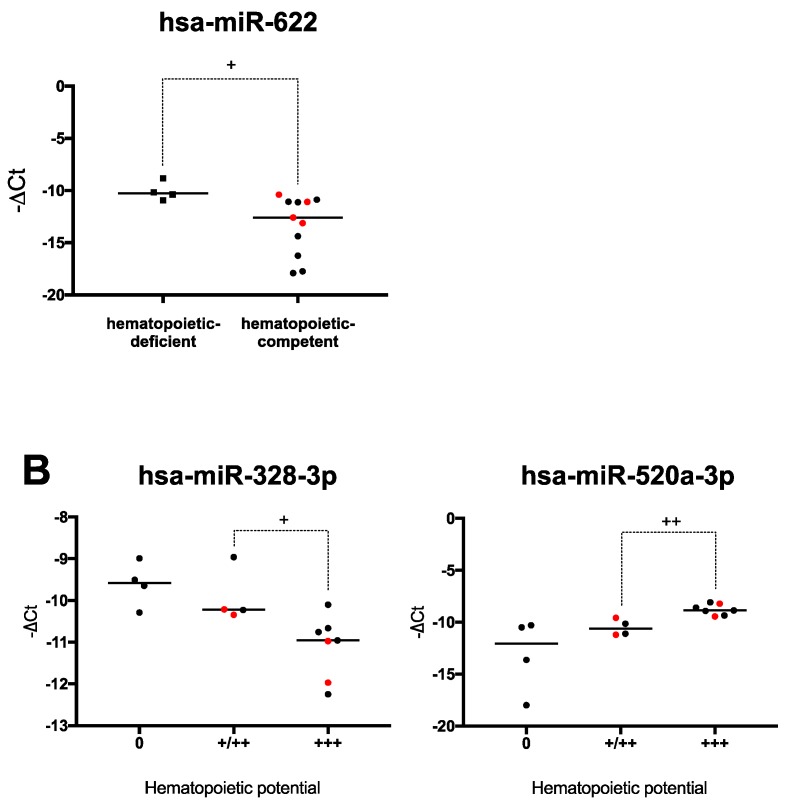
Differential miRNA expression according to hPSC hematopoietic capability. (**A**) The expression level of miR-105, miR-122, miR-135b, miR-206, miR-296, miR-335, miR-492, miR-515, miR-520a and miR-622 in hematopoietic-deficient and -competent hPSCs, at the pluripotent stage, is shown as vertical scatter dot plot with median; (**B**) The expression level of miR-328 and miR-520a in hematopoietic-deficient, poor and high competent hPSCs, at the pluripotent stage, is shown as vertical scatter dot plot with median. MicroRNA expression levels were estimated by a –ΔCt calculation (with ΔCt = Ct miRNA – Ct RNU48), from the TLDA analysis. Human ES cells are represented in red and hiPSCs in black. P-values were calculated by using the LiMMa package from Integromics (+ *p* < 0,05; ++ *p* < 0,01; +++ *p* < 0,001).

A second supervised study comparing miRNA expression between hematopoietic-deficient and -competent hPSCs was performed. TLDA results were analyzed by the LiMMa moderate t-test. Using this approach, 10 miRNAs out of 754 appeared significantly differentially expressed (Table S4, ES + iPS, statistical significance in the table). It should be noted that these differences were also significant when tested using a two-tailed Mann-Whitney U-test. Overall, we found a lower expression level of miR-206, miR-135b, miR-105, miR-622, and miR-492 in hematopoietic-competent hPSCs (Figure 3A). In contrast, the expression of miR-520a, miR-296, miR-122, miR-515, and miR-335 was higher in these cells. Two miRNAs belonging to the large miRNA cluster C19MC located on human chromosome 19 (miR-515 and miR-520a) displayed significantly increased expression in hematopoietic-competent cells. Of note, miR-206 was the only miRNA of which the differential expression remained significant after LiMMa moderate t-test and Bonferroni correction (*p* < 0.01).

Since the four hematopoietic-deficient cell lines used in our study were from iPS cells, we removed data from the four hES cell lines and performed the same statistical analyses on the remaining 11 hiPS cells. In this situation, underexpression of miR-206, miR-105, miR-622, and an over-expression of miR-122, miR-520a, miR-296, miR-335, miR-106a (not detected in the global hPSC testing) were observed in hematopoietic-competent hiPSCs (Table S4, iPS alone). As previously observed miR-206 retained significant differential expression after Bonferroni correction (*p* < 0.01).

Finally, we tested whether some specific miRNAs could allow us to discriminate between poor and high hematopoietic-competent hPSCs (as defined in Table 2). Among the 11 miRNAs identified by the LiMMa method, only miR-520a remained significantly upregulated after Bonferroni correction (Table S5, statistical significance in the table). Moreover, miR-520a and miR328 displayed gradual expression patterns from hematopoietic-deficient to hematopoietic-competent cell lines going through an intermediary level correlated with intermediate or poor hematopoietic potential (Figure 3B).

### 2.7. Integrative miRNome and Transcriptome Analysis

**Figure 4:**
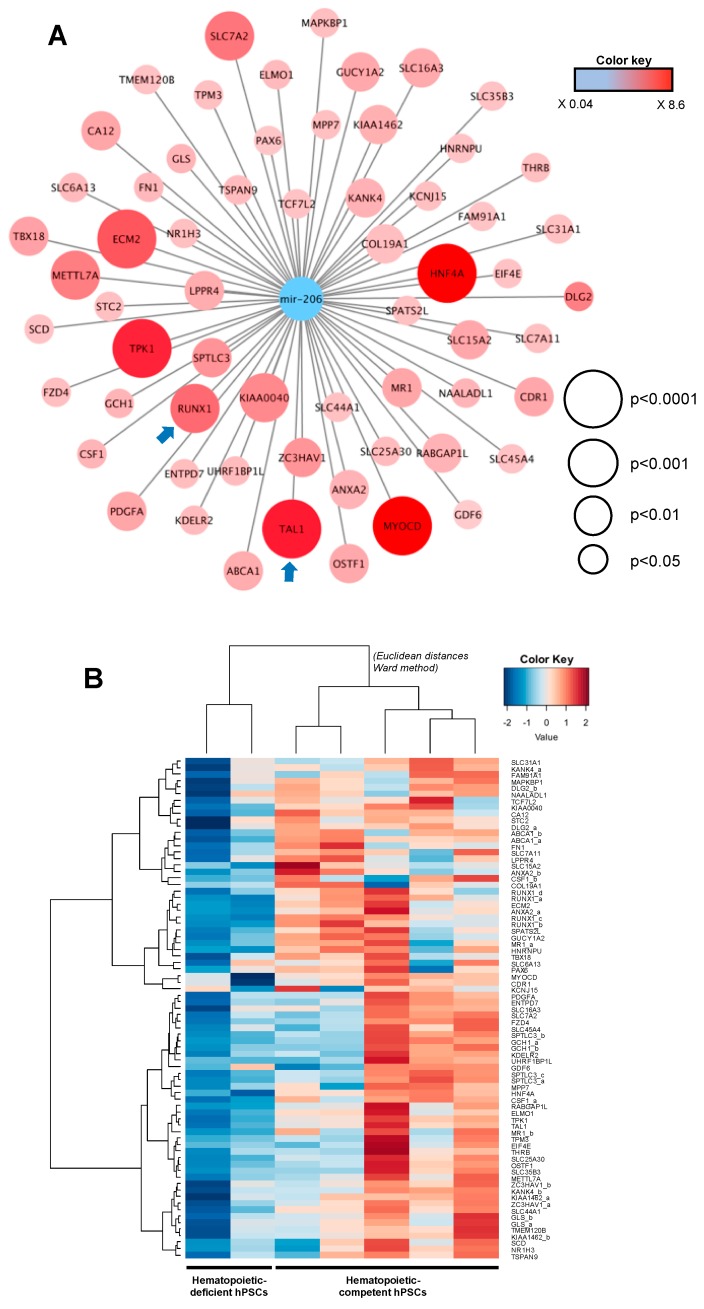
Integrative miRNome and transcriptome analysis. (**A**) Cytoscape representation of a predictive miR-206-regulated gene network potentially involved in the regulation of the hematopoietic differentiation of human pluripotent stem cells; (**B**) Unsupervised expression heatmap of 62 miR-206 target genes predicted in TargetScan v7.2 distinguishes EB cells according to their hematopoietic potential. Blue arrows highlight hematopoietic master regulators RUNX1 and TAL1.

Given that miR-206 displayed the most significant downregulation in hematopoietic-competent hPSCs, we conducted an integrative bioinformatic analysis on miR-206 predicted target genes. To this end, 773 mRNA target transcripts of the broadly conserved (across vertebrates) miR-1-3p/206 family were identified in the TargetScan database and integrated into the global transcriptomic analysis performed by microarray on day-16 EBs. Using supervised ranking product analysis, 62 predicted genes and targets of the miR-1-3p/206 family were found significantly up-regulated in hematopoietic-competent samples (Figure 4A, Table S6), including the hematopoietic transcription factors *RUNX1* and *TAL1*. Hierarchical unsupervised clustering, based on this subset of 62 predicted mir-206 target genes, fully discriminated hematopoietic-deficient from -competent cells (Figure 4B).

Additional qRT-PCR experiments were carried out to explore the expression of both miR-206 and *RUNX1*/*TAL1* mRNA in a subset of hematopoietic-competent hPSCs and their respective day-16 EBs (Figure S5). As expected, low levels of *RUNX1* transcripts were detected at the pluripotent stage, while *TAL1* transcripts were mostly undetectable. However, at the day-16 EB stage, a significant increase in both *RUNX1* and *TAL1* mRNA levels was observed. In addition, at the pluripotent and day-16 EB stages, miR-206 expression remained very low.

### 2.8. Overexpression of miR206 in the H1 ES Cell Line

**Figure 5:**
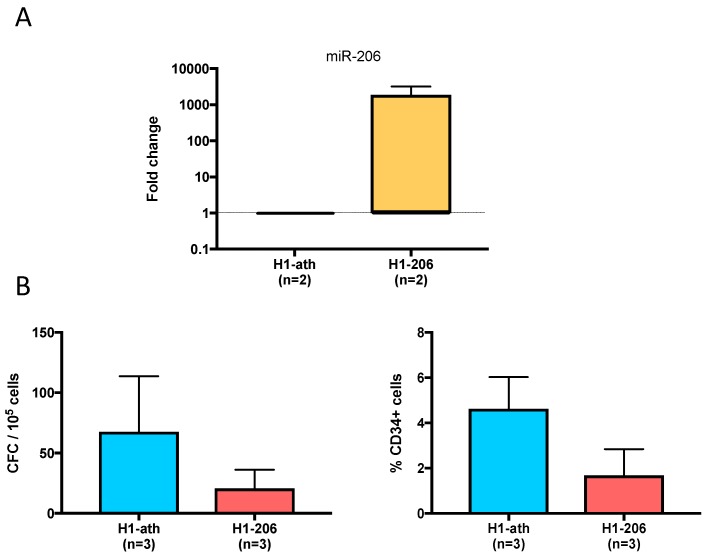
Inhibition of the hematopoiesis process by overexpression of miR-206 in a blood-competent ES cell line. The hematopoietic-competent H1 ES cell line was transduced with a lentivirus expressing either miR-206 (H1-206) or the ath-miR-159a negative control (H1-ath). (**A**) miR-206 average expression in H1-206 cells as compared to H1-ath cells. Fold changes were calculated by the 2^−ΔΔCt^ method with RNU48 as endogenous control; (**B**) The number of CFCs and the percentage of CD34+ cells in day-16 EBs (mean ± SD) are shown on the graphs for H1-ath control (blue bar) and H1-206 (red bar).

In our analysis, miR-206 appeared to be highly repressed in hematopoietic-competent hPSCs. To test the relevance of this miRNA in the regulation of early fetal hematopoiesis, we transduced the H1 hematopoietic-competent ESC line with a vector expressing either miR-206 (H1-206) or the unrelated *Arabidopsis thaliana* (ath)-miR-159a (H1-ath) as a control. As shown in Figure 5A, lentiviral transduction resulted in >1000-fold overexpression of miR-126 in undifferentiated H1 cells. In three independent experiments, a trend towards a decrease of both the hematopoietic colony number and the percentage of CD34-positive cells was observed in H1-206-derived day-16 EBs as compared to H1-ath counterparts (Figure 5B). These results suggest that miR-206 could act as a repressor of hematopoietic differentiation in hPSCs.

## 3. Discussion

We and others have previously shown that several hPSC lines were heterogeneous with variable hematopoietic potential, assessed by in vitro clonogenic assay and CD34+ cell count in EB cells [10,16] and whole transcriptome analysis performed on day-16 EBs. The mRNA expression of *CD34* adhesion molecule, *TAL1 (SCL)*, *GATA1*, *RUNX1*, *SPI1/PU.1* transcription factors were significantly higher in hematopoietic-competent PSCs as compared with hematopoietic-deficient cells. Similarly, germ layer-related genes involved in mesodermal differentiation, such as *KDR*, *HAND1*, and *COL1A1*, were upregulated in hematopoietic-competent PSCs. The objective of this work was to identify miRNAs of which the expression in hPSCs at the pluripotent stage could predict their capacity to differentiate into the hematopoietic lineage.

In our previous study, we concluded that the origin of hPSCs represented a key determinant of variation in their hematopoietic potential [16]. In the present study, most of the hematopoietic-deficient iPSCs originated from Down syndrome amniocentesis (3/4) and had an extra copy of chromosome 21. It has been reported that trisomy 21 could modify the fetal hematopoietic differentiation process [18]. Along these lines, human trisomy 21 PSCs were shown to display either increased erythropoiesis and a reduced myelopoiesis [19], or an increase in all hematopoietic progenitor populations [20]. Therefore, although trisomy 21 can modify the hematopoietic differentiation in hPSCs, it does not abrogate it, thereby rendering our model suitable for predicting miRNA signatures during hematopoietic differentiation of hPSCs. It should also be mentioned that all miRNAs identified in our study are located outside of chromosome 21 and that one of our hematopoietic-competent iPSC lines also had trisomy 21.

Ramos-Mejia et al. suggested that the NODAL/ACTIVIN signaling pathway could distinguish pluripotent stem cells according to their hematopoietic differentiation potential [10]. The authors showed that upregulation of some members of the NODAL/ACTIVIN signaling pathway at the pluripotent stage improved the hematopoietic potential of hES cells. Here, we measured in our panel of hPSC lines the mRNA expression of different members of the NODAL/ACTIVIN pathway and found no significant differential expression for any of these genes between hematopoietic-deficient and -competent hPSCs at the pluripotent stage. This discrepancy may be attributed to the fact that most of the PSCs investigated in the present study are of iPSC nature. In this case, reprogramming may introduce some variations in developmental processes in comparison to hESCs. In addition, we analyzed the ability of hPSCs to give rise to blood cell progenitors, while Ramos-Mejia et al. investigated variations in the hematopoietic potential.

For hPSCs lacking blood differentiation potential, we hypothesized that the absence of hematopoietic progenitors in day-16 EBs might be reminiscent of an early defect in the onset of the hematopoiesis differentiation program. The importance of miRNAs in hematopoiesis is now well established. Their expression in the hematopoietic stem and progenitor cells and in the medullar microenvironment plays a critical role in blood cell differentiation [21]. To test our assumption, we focused on five blood-related miRNAs (miR-125b, miR-142-3p, miR-150, miR-155, miR-223). We measured their expression in EBs undergoing hematopoietic differentiation at 0, 3 and 16 days of culture. Day-0 samples corresponded to the pluripotent stage while day-3 EBs host the primitive bipotent hemato-endothelial progenitor hemangioblast [22]. We found no significant differences in miRNA expression between iPSCs and ESCs whatever their hematopoietic potential. This suggests that the reprogramming process did not particularly alter the expression of these miRNAs and did not introduce a bias in the study. As expected, miR-302 expression level decreased progressively upon hematopoietic differentiation, while that of miR-125b increased starting at the hemangioblast stage from which HSCs are thought to emerge. MicroRNAs miR-302 and miR-125b are enriched in human embryonic stem cells and CD34+ hematopoietic stem/progenitor cells, respectively [23,24]. Blood-specific miR-223 appeared largely up-regulated along the culture period, thereby confirming the activation of the hematopoiesis differentiation program. The reduced expression of miR-223 and miR-142 in the hematopoietic-deficient PB9 iPS cell line at the 3rd day of culture highlights the importance of these two miRNAs in the early stage of hematopoietic differentiation. Indeed, miR-223 is involved in myeloid commitment and regulation of myeloid cell lineage [25], whereas miR-142 is expressed in all blood cells, is essential for hematopoietic development in zebrafish [26] and mouse [27], and has been shown to be critical to specification of the hemangioblast in a Xenopus model [28]. These data could suggest a defect in the activation of the blood differentiation program during EB culture of PB9 cells, which could result in reduced expression of key transcription factors and miRNAs in day-16 EBs, hence their inability to generate hematopoietic progenitor cells. However, the kinetics of miR-223 and miR-142 expression in the second hematopoietic-deficient iPS cell line tested (PB6) suggests that the deficiency in blood progenitor generation seems unrelated to the ability at expressing these miRNAs. More generally, low global expression levels of miR-142, miR-150, miR-155, and miR-223 hematopoietic-specific miRNAs in day-3 EBs do not appear to be good predictors of poor hematopoietic potential.

Overall miRNome analysis is now widely used to identify novel biomarkers for diagnosis, prognosis, or response to therapy in cancer diseases [29]. MicroRNA expression data can also be used to predict the behavior of living cells in particular experimental or physiological conditions. To identify novel biomarkers of PSCs’ hematopoietic potential, we conducted a miRNA expression profiling analysis of 15 hPSC lines at the pluripotent stage. Statistical analyses revealed significant differences in the expression level of several miRNAs between hematopoietic-deficient and -competent PSCs. Surprisingly, the most upregulated miRNA in blood-competent hPSCs was the liver-specific miR-122, both when performing the analysis on all hPSCs or on hiPS cells only. MicroRNA-122 was shown to regulate iron homeostasis and to exert tumor suppressor activity inhibiting the formation of hepatocellular carcinoma [30,31]. In vitro experiments underlined the role of miR-122 in the hepatic differentiation of fetal liver-derived stem/progenitor cells [32]. Expression of miR122 in blood-competent hPSCs could link EB-derived hematopoiesis to fetal liver hematopoiesis, where definitive hematopoiesis takes place during embryonic development [33]. Increased expression of two members of the C19MC cluster (miR-515 and miR-520a) was also observed in hematopoietic-competent cells. High expression of the C19MC cluster, together with the miR-302 cluster, is a characteristic of undifferentiated hES cells [34,35]. However, only miR-520a retained significant differential expression when analyzing hiPS cells only. MiR-520a was also significantly upregulated in PSCs with high hematopoietic potential as compared to those with poor blood differentiation capacity. The expression of miR-520a increased with hematopoietic potential, while that of miR-328 decreased. Other miRNAs displayed differential expression between the 2 PSC groups. Although not having been described to play a role in hematopoiesis, higher expression of miR-335 and miR-296 was also seen in blood-competent cells. MicroRNA miR-335 was shown to exert oncogenic activity while miR-296 acts as a tumor suppressor in tumor models [36,37]. On the other hand, some miRNAs displayed lower expression in the blood-competent group, including miR-622 and miR-105, which has been shown to play a role in megakaryopoiesis [38].

Among miRNAs highlighted in our work, miR-206 displayed the strongest downexpression in hematopoietic-competent cells. Furthermore, it remained the only miRNA significantly differentially expressed after LiMMa moderate *t*-test with Bonferroni correction, both in the entire panel of PSCs and in the panel restricted to iPSCs. Non-coding RNAs (such as miRNAs) play a critical role in the development of cancer. In this context, decreased miRNA expression could reduce cellular differentiation [39]. More specifically, it has been shown in a breast cancer model that miR-206 could target cancer stem cells by inhibiting their self-renewal [40].

The broadly conserved miR-1-3p/206 family has not been involved in hematopoietic differentiation but is essentially expressed in mesoderm-derived skeletal and cardiac muscle cells [41,42]. This could suggest that elevated miR-206 levels may inhibit hematopoiesis commitment of hPSCs by promoting muscle cell differentiation programs. Canonical myomiRs (miR-1, -133 and -206) act as key components of the development of skeletal and cardiac muscles, even though they could also fulfill regulatory functions in non-muscular tissues [43]. Indeed, it has been shown that overexpression of the miR-206 paralog miR-1 in ES cells promoted mesoderm formation and muscle cell differentiation while repressing non-muscle gene expression [44]. Interrogation of the TargetScan database identified 773 genes containing predicted conserved sites for miR-1-3p/206 binding. They were integrated into global transcriptomic analysis, and 62 predicted miR-206 target genes were found to be significantly up-regulated in hematopoietic-competent EBs. Most of them are not known to exert any function in the hematopoietic differentiation process. An explanation could be provided by the fact that the transcriptome experiment was performed on day-16 EBs and not at the pluripotent stage. Nevertheless, two potential targets of miR-206, *RUNX1* and *TAL1* appeared significantly over-expressed in hematopoietic-competent EBs. These two transcription factors are well-known critical regulators of hematopoiesis [45,46]. Moreover, we showed that the expression of *RUNX1* and *TAL1* mRNAs was significantly increased during hematopoietic differentiation, whereas the expression of miR-206 remained at low level. Although not the initial purpose of our work, a link between miR-206 downregulation and *RUNX1*/*TAL1* mRNA expression in hematopoietic-competent hPSCs can be suggested. However, the expression of miR-206 target genes does not fully explain its complete role in the regulation of hematopoietic potential of hPSCs, and the direct targeting of *RUNX1* and *TAL1* by miR-206 remains to be demonstrated. Nevertheless, the reduction in hematopoiesis induced by the enforced expression of miR-206 in H1 hematopoietic-competent ES cells supports miR-206 as a potent inhibitory regulator of hPSCs hematopoietic differentiation.

## 4. Materials and Methods

### 4.1. Cell Lines and Culture

Human iPSCs were generated at the Pluripotent Stem Cell Core Facility (ESTeam Paris Sud, Inserm U935, Villejuif, France; esteamparisud.fr). Details of the reprogramming methods are available upon request. All cell lines were extensively characterized throughout the study and at multiple passages during processing and banking. Tests included phenotyping, cytogenetics, and teratoma formation assay in NOD-SCID mice. The human ESC lines used in the present study (Authorization number RE10-035R/C from Biomedicine Agency, Saint-Denis, France) were SA01 (Cellartis, Göteborg, Sweden), H1 and H9 (WiCell Research Institute Inc, Madison, WI, USA), and CL01 derived in our laboratory (described in the Human Pluripotent Stem Cell Registry, www.hescreg.eu). All hPSCs were expanded and stored in the Pluripotent Stem Cell Core Facility.

Cells were cultured on mitomycin C (20 mg/mL)-inactivated mouse embryonic fibroblasts in KnockOut DMEM medium supplemented with 20% Knock-Out Serum Replacement, 1 mM L-glutamine, 100 mM 2-mercaptoethanol, 0.5% penicillin/streptomycin (Invitrogen, Carlsbad, CA, USA) and 5 ng/mL bFGF (Miltenyi Biotech, Bergisch Gladbach, Germany). Half of the medium was changed daily, and cells were passaged weekly with collagenase type IV (Invitrogen). Cultures were incubated in a humidified atmosphere at 37 °C, 5% CO2.

### 4.2. Differentiation of hPSCs in EB Cells

The small clumps obtained after collagenase treatment were cultured in Iscove’s modified Dulbecco’s medium (IMDM, Invitrogen), supplemented with 15% Fetal Calf Serum, 450 µM monothioglycerol, 50 µg/mL ascorbic acid, 200 µg/mL transferrin (Sigma Aldrich, St-Louis, MO, USA), 100 ng/mL of Stem Cell Factor and FLT3-L and 50 ng/mL thrombopoietin (PeproTech, Neuilly-sur-Seine, France). The medium was changed 2–3 times along the 16-day culture period, depending on EB proliferation. All cultures were incubated in a humidified atmosphere at 37 °C, 5% CO2.

### 4.3. Clonogenic Progenitor Assays

Cells were harvested from day-16 EBs by mechanical dissociation with gentle pipetting in phosphate buffered saline (PBS) and plated in triplicate at 5 × 10^4^ or 10^5^ cells/mL into MethoCult GF H84435 (StemCell Technologies, Vancouver, BC, Canada). Hematopoietic colonies (CFU-E, BFU-E, CFU-GM, and CFU-GEMM) were then counted at day 14.

### 4.4. Flow Cytometry

Single-cell suspensions were incubated with the following fluorochrome-conjugated antibodies: CD31-FITC, CD43-FITC, CD34-APC, CD45-PE, CD235-APC, CD41-PE, CD36-PE, CD71-PE (BD Biosciences, San Jose, CA, USA; Beckman Coulter, Brea, CA, USA). Cells were incubated for 30 min on ice, washed and resuspended in PBS containing 5% Fetal Calf Serum and 1 µg/mL 7-amino actinomycin D (7-AAD, Sigma Aldrich) to exclude dead cells. The analysis was carried out with a FACSCalibur flow cytometer (BD Biosciences).

### 4.5. Total RNA, miRNA Extraction, and cDNA Synthesis

Total RNA was extracted using Trizol according to the manufacturer’s recommendations (Invitrogen), treated with DNase I (Invitrogen), poly-adenylated using *E. coli* poly(A) polymerase (Ambion, Carlsbad, CA, USA), and finally reversed-transcribed using SuperScript III (Invitrogen) and standard oligo-d(T) or the 5’-GCGAGCACAGAATTAATACGACTCACTATAGGAC-GGCTTTTTTTTTTTTTTTVN-3’ oligo-d(T)/adapter primer.

### 4.6. Whole Transcriptome and qRT-PCR Analyses

Human Agilent whole transcriptome analysis was carried out on total RNA extracted from embryonic body samples. Background-subtracted green fluorescent signals generated using Feature Extraction software (Agilent Technologies, Santa Clara, CA, USA) were merged into a normalized matrix for all the tested samples. Log2-base transformation of the normalized signal was computed after filtration of accepted control probes spotted on Agilent microarray. Agilent gene identifier was annotated with Gene Expression Omnibus platform GPL10332 (Agilent Whole Human Genome Microarray 4x44K v2) obtained on NCBI website (http://www.ncbi.nlm.nih.gov/geo). The mRNA level of selected genes encoding proteins involved in the NODAL/ACTIVIN signaling pathway was also determined by qRT-PCR (Table S2). Real-time PCR experiments were performed using a standard process and *GAPDH* as the reference gene.

### 4.7. Hematopoiesis-Related miRNA qRT-PCR Analysis

MicroRNA qRT-PCR was performed using 1 µL of 1/10-diluted cDNA in a 10-µL reaction containing 0.3 µM of the universal adapter-specific reverse primer 5’-GCGAGCACAGAATTAATACGACTCAC-3’, the miRNA-specific forward primer (Table S7), and 1X FastStart SYBR Green MasterMix (Roche Diagnostics, Meylan, France). Endogenous control RNU48 was amplified using 5′-TGATGATGACCCCAGGTAACTC-3′ (forward) and universal reverse primer. Real-time PCR was performed in triplicate reactions on a Mx3000P qPCR system (Agilent Technologies), with the following settings: 95 °C/10min, then 45 cycles of 95 °C/15s, 60 °C/1min, followed by dissociation cycle.

### 4.8. TLDA Analysis

Reverse transcription reaction was performed using the human Megaplex™ primer pools set v3.0 (Life Technologies, Foster City, CA, USA), which contains RT primers for 754 individual miRNAs, according to manufacturer’s instructions. Real-time qRT-PCR was then carried out on an ABI 7900HT real-time PCR machine with the LDA thermal cycler block, using TaqMan^®^ array human microRNA card set v3.0 (Life Technologies) and pre-defined TLDA thermal cycling conditions. Quantitative RT-PCR data were extracted with SDS2.3 and RQ Manager Software (Life Technologies). Thresholds for the determination of Ct values were manually set at 0.2, 0.1, or 0.05 for each miRNA across the 15 samples, depending on the quality of the amplification. Relative fold change was then calculated using the ΔCt method, normalized to RNU48 expression.

### 4.9. Integrative miRNome and Transcriptome Analysis

Predicted target genes of miR-206 were retrieved from the TargetScan database (release 7.2, March 2018) [47]. They were then merged with the Agilent transcriptome matrix. Microarray supervised ranking product analysis was performed with MeV 4.9.0 software [48]. Bioinformatics analysis was conducted in R programming environment version 3.4.3. Expression heatmap was performed with R Bioconductor package made4 [49]. Integrative networks were obtained with network software Cytoscape version 3.6.0 [50].

### 4.10. Overexpression of miR-206 in a Blood-Competent ES Cell Line

The miR-206 over-expressing vector was prepared using a modified pLKO.1 lentiviral plasmid (Sigma Aldrich) containing an eGFP-2A-Puro cassette (pLKO-G2AP, kind gift from C. Bailey and J. Rasko, Centenary Institute, Sydney, Australia). A region of 413 bp containing the pre-hsa-miR-206 DNA sequence was PCR-amplified using forward: 5′-CTGCCATTCCTCACAACAGA-3′ and reverse: 5′-CTCAAGAGGGGGAGATAGGG-3′ primers and cloned into the AgeI and XbaI sites of pLKO-G2AP. Control plasmid consisted in an *Arabidopsis thaliana* microRNA sequence (ath-miR-159a), which has no specific targets in mammalian cells. This sequence was also cloned in a modified pLKO.1 vector (pLKO-E2AP, Centenary Institute, Sydney, Australia).

Viral particles were obtained by calcium phosphate transfection of the 293FT cell line with a combination of pLKO vector, packaging and pseudotyping plasmids (Rev, ΔR, VSVG). H1 cells were transduced with 250 µL ES medium containing 2.5 µL concentrated viral supernatant and 8 µg/mL protamine sulfate and incubated overnight, 37 °C, 5% CO2. Transduced cells were then selected by incubation with 0.3 µg/mL puromycin, which was maintained for stable clone expansion. The proportion of H1 cells expressing eGFP was controlled by flow cytometry (85.84% for the H1-ath control and 87.25% for the H1-206). In addition, the overexpression of ath-mir-159 in H1-ath and mir-206 in H1-206 was assessed by qRT-PCR.

### 4.11. Statistical Analysis

TLDA data were analyzed with StatMiner Software (Integromics, Armilla, Spain). To identify differentially expressed target miRNAs, the LiMMa (Linear models for microarrays) moderate *t*-test was used adjusting or not the p-value with the Bonferroni correction. TLDA and qRT-PCR data were also analyzed with the nonparametric 2-tailed Mann-Whitney U-test applied to the ΔCt values. Results with a *p*-value < 0.05 were considered as statistically significant. Statistical tests and graphical representations were carried out using GraphPad Prism version 7.0 (GraphPad Software, La Jolla, CA, USA).

## 5. Conclusions

Pluripotent stem cells (ESCs and iPSCs) display some variations in their hematopoietic differentiation ability. In this work, we identified several miRNAs (miR-206, miR-520a, miR-296, miR-122, miR-335, miR-105, miR-622) that may prove useful in prediction of the hematopoietic potential of hPSCs at the pluripotent stage. MiR-206, classically involved in mesoderm-derived muscle development, appears critical to regulation of hematopoietic differentiation of hPSCs since its overexpression in a blood-competent ES cell impairs this process. The complete characterization of miR-206 and its potential target gene network as regulators of hematopoietic differentiation warrants further studies since the functions of most of these genes are poorly understood in this context. Overall, this work suggests a significant role of miR-206 as a negative regulator of hematopoietic commitment and highlights its usefulness as a biomarker for identifying hematopoietic-competent human pluripotent stem cells.

## Figures and Tables

**Table 1:** Human pluripotent stem cell (hPSC) lines used in this work.

Cell Line	Passage	Karyotype/CGH	Origin
**iPSC lines**			
PB3	43	46, XX	Normal amniocyte
PB4	26	46, XY	Amniocyte, sickle cell anemia
PB6	23	47, XY, +21	Amniocyte, Down syndrome
PB6.1	14	47, XY, +21	Amniocyte, Down syndrome
PB7	63	46, XY	Normal amniocyte
PB9	17	46, XY	Normal amniocyte
PB10	16	46, XY	Normal amniocyte
PB12.1	33	47, XY, +21	Amniocyte, Down syndrome
PB13	28	46, XY	Endothelial progenitor cell from peripheral blood
PB14.3	41	47, XX, +21	Amniocyte, Down syndrome
PB17	38	46, XY	Endothelial progenitor cell from peripheral blood
**ESC lines**			
SA01	22+4+7	46, XY	Cellartis AB, Sweden
H1	67	46, XY	WiCell, Wisconsin, USA
H9	31	46, XX	WiCell, Wisconsin, USA
CL01	16	46, XY, der21, t(1;21)	Paris Sud University, France

All iPSC lines were generated in ESTeam Paris Sud University, Inserm U935, Villejuif, France. CGH, Comparative Genomic Hybridization; der21, t(1;21), unbalanced chromosome translocation involving chromosome 1 and chromosome 21.

**Table 2:** Characterization of the hematopoietic potential of the hPSC lines used in this work.

Cell line		CFC/10^5^ Cells	*Distribution of Hematopoietic Colony Subtypes (/10^5^ cells)*				%CD34^+^ Cells	Hematopoietic Potential
			*CFU-E*	*BFU-E*	*CFU-GM*	*CFU-GEMM*		
iPSCs	PB3	140	*0*	*1*	*139*	*0*	35 ± 10	+++
	PB4	80	*0*	*1*	*79*	*0*	13 ± 3	++
	PB6	2	*1*	*0*	*1*	*0*	2 ± 1	0
	PB6.1	120	*41*	*46*	*30*	*3*	17 ± 7	+++
	PB7	144	*52*	*33*	*58*	*1*	13 ± 6	+++
	PB9	0	*0*	*0*	*0*	*0*	0.3	0
	PB10	24	*0*	*4*	*20*	*0*	3 ± 0.3	+
	PB12.1	0	*0*	*0*	*0*	*0*	0.4	0
	PB13	249	*115*	*134*	*0*	*0*	16 ± 10	+++
	PB14.3	5	*0*	*1*	*4*	*0*	3 ± 0.5	0
	PB17	235	*47*	*0*	*188*	*0*	17 ± 4	+++
ESCs	SA01	402	*145*	*84*	*161*	*12*	23 ± 9	+++
	H1	318	*216*	*29*	*70*	*3*	33 ± 16	+++
	H9	38	*2*	*1*	*35*	*0*	9 ± 2	++
	CL01	41	*2*	*1*	*37*	*1*	8 ± 2	++

CFC, colony-forming cell; CFU-E, colony-forming unit-erythroid; BFU-E, burst-forming unit-erythroid; CFU-GM, colony-forming unit-granulocytes macrophages; CFU-GEMM, colony-forming unit-granulocytes erythrocytes macrophages megakaryocytes. Concerning hematopoietic potential, 0 represents iPS cell lines lacking hematopoietic differentiation ability, +/++ poor hematopoietic-competent hPSCs (less than 100 colonies/10^5^ cells), +++ high hematopoietic-competent hPSCs (more than 120 colonies/10^5^ cells).

## Supplementary Materials

Supplementary Materials can be found at https://www.mdpi.com/1422-0067/20/7/1737/s1.

## Abbreviations

ath	Arabidopsis thaliana
BFU-E	Burst-forming unit-erythroid
CFC	Colony-forming cell
CFU-E	Colony-forming unit-erythroid
CFU-GEMM	Colony-forming unit-granulocytes erythrocytes macrophages megakaryocytes
CFU-GM	Colony-forming unit-granulocytes macrophages
CGH	Comparative genomic hybridization
EB	Embryoid bodies
ESCs	Embryonic stem cells
hiPSCs	Human induced pluripotent stem cells
hPSCs	Human pluripotent stem cells
LiMMa	Linear models for microarrays
miRNAs	Micrornas
PSCs	Pluripotent stem cells
qRT-PCR	Quantitative real-time RT-PCR
TLDA	TaqMan low-density array
