# Supplementary material for: Global MicroRNA Profiling Uncovers miR-206 as a Negative Regulator of Hematopoietic Commitment in Human Pluripotent Stem Cells
Source: Int J Mol Sci. 2019 Apr 8;20(7):1737. doi: 10.3390/ijms20071737 (PMC6479521; doi:10.3390/ijms20071737)
Supplement: Supplementary file 1 [file ijms-20-01737-s001.pdf]

Supplementary Materials

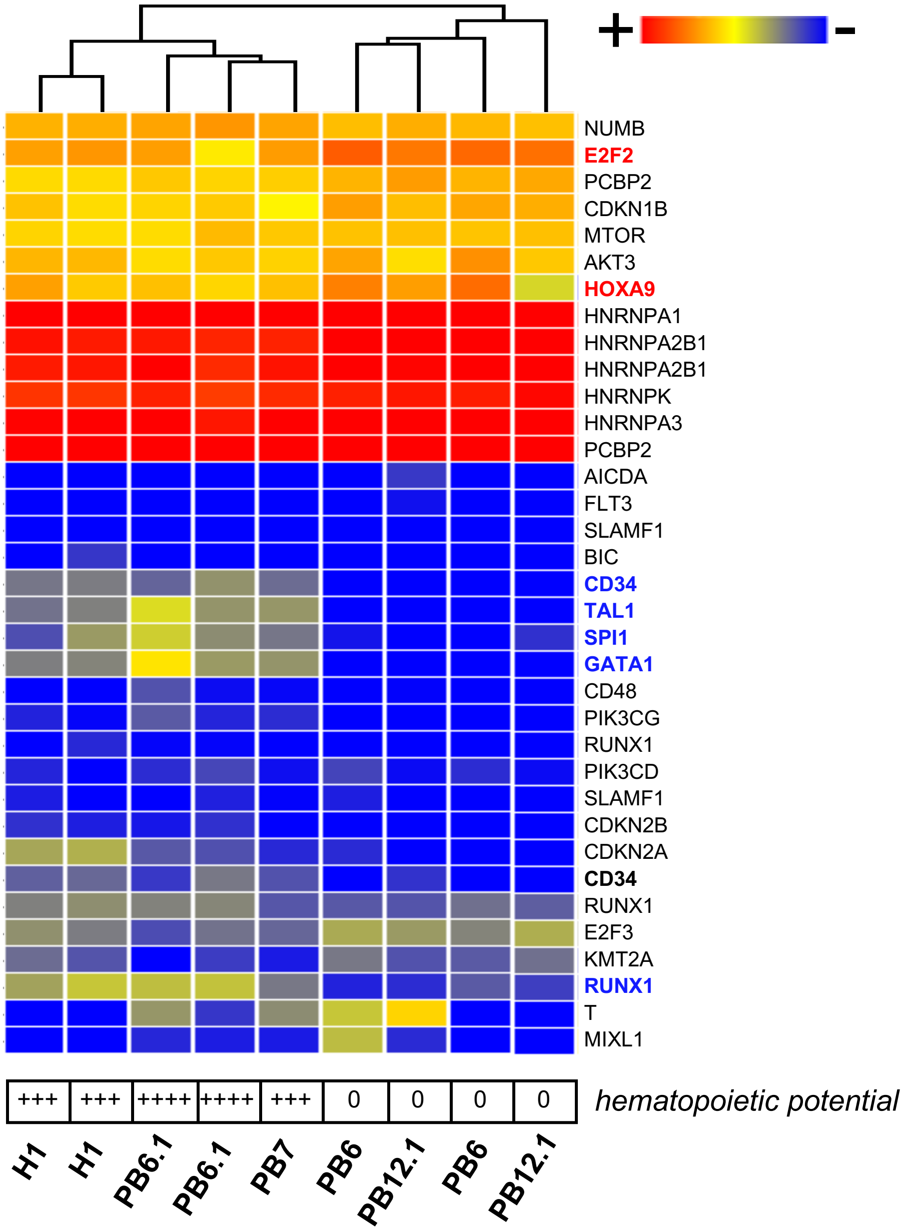

Figure S1. Unsupervised hierarchical clustering of hPSC-derived EBs according to the mRNA expression of hematopoietic lineage genes (microarray analysis). Hematopoietic-competent cells (H1, PB6.1, PB7) were separated from hematopoietic-deficient ones (PB6, PB12.1). In this experiment, all hPSCs were tested in duplicate, except PB7. Genes under-expressed or over-expressed in blood-deficient hPSCs are indicated in blue and red respectively (*related to Table S1*).

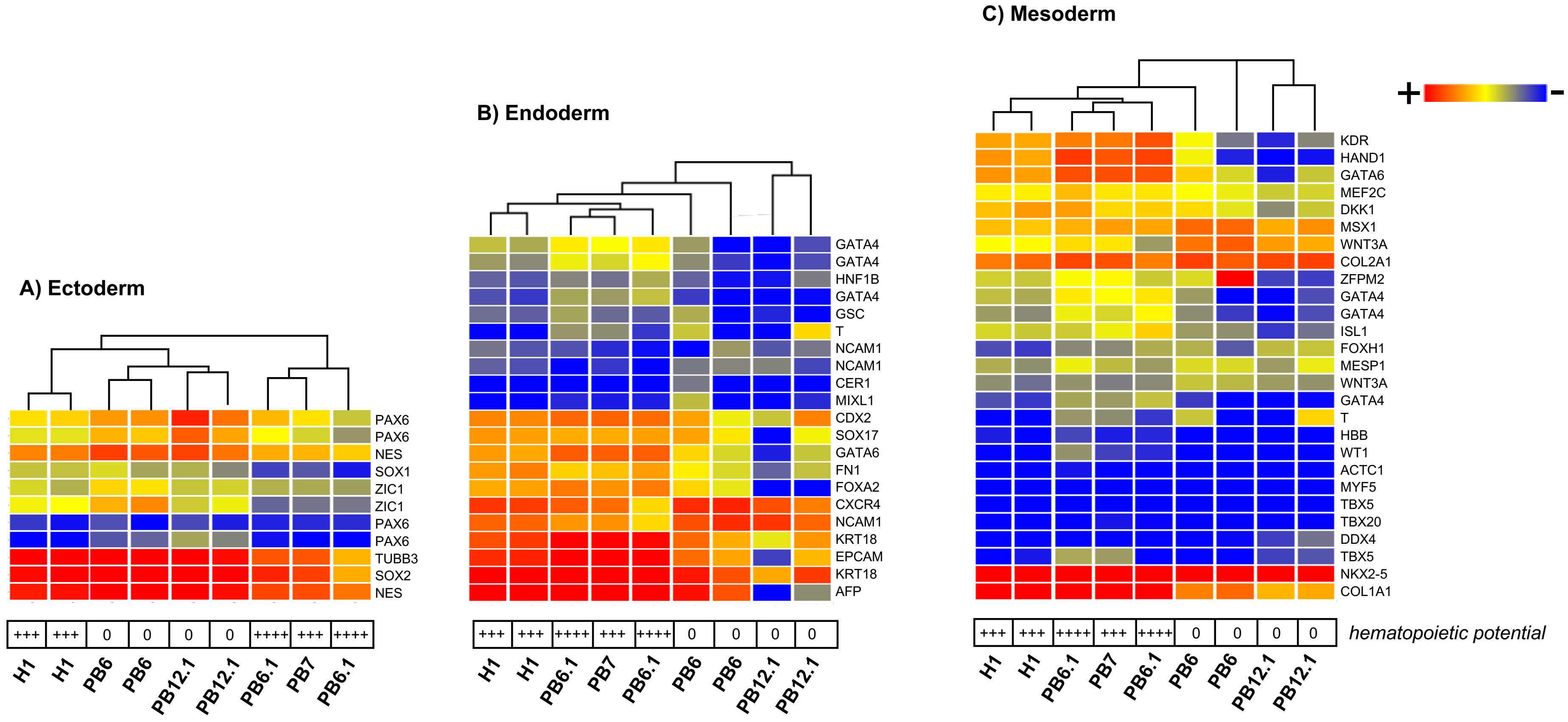

Figure S2. Unsupervised hierarchical clustering of hPSC-derived EBs according to the mRNA expression of germ layer differentiation genes (microarray analysis) Selected ectoderm (A), endoderm (B) and mesoderm (C) related genes differentially expressed between hematopoietic-competent (H1, PB6.1, PB7) and -deficient cells (PB6, PB12.1) are shown (r*elated to Table S1).*

A) NODAL/ACTIVIN family schematic pathway

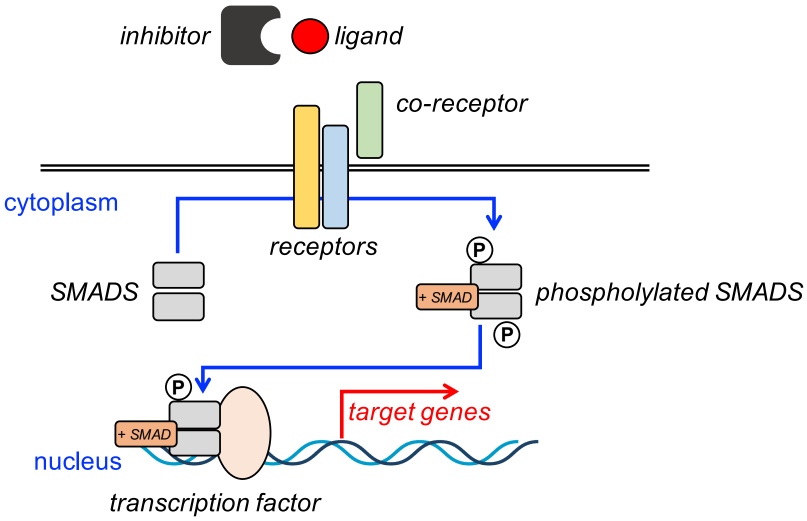

B) Actors of NODAL/ACTIVIN signaling pathway

Ligand	Receptor	Co-receptor	Inhibitor	Signal transduction factor	Additional transcription factor
**ACTIVIN** (1)	**ACVR1B** (1) (ALK4), **ACVR1C** (1) (ALK7) / **ACVR2A** (1) (ACTR2A), **ACVR2B** (1) (ACTR2B)		**FST** (1) (FOLLISTATIN)	SMAD 2, 3 (+ SMAD 4)	**FOXH1** (2)
**NODAL** (1,2)	**ACVR1B** (1) (ALK4), **ACVR1C** (1) (ALK7) / **ACVR2A** (1) (ACTR2A), **ACVR2B** (1) (ACTR2B)	**CRIPTO** (1,2) (TDGHF1)	**CER1** (1,2) (CERBERUS), **LEFTY1** (1,2), **LEFTY2** (1,2)	SMAD 2, 3 (+ SMAD 4)	**FOXH1** (2)
BMPs (**BMP2** (1), **BMP4** (1), …)	**BMPR2** (1) / **BMPR1A** (1) (ALK3), **BMPR1B** (1) (ALK6)			SMAD 1,5,8 (+ SMAD 4)	

C) Microarray analysis in day-16 EB cells

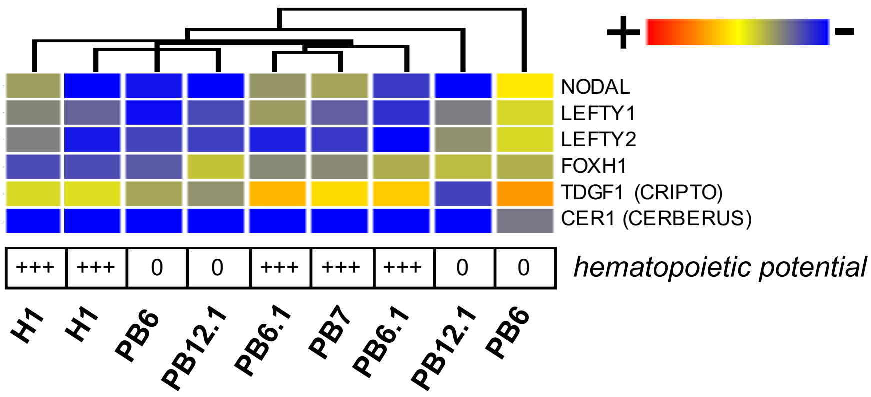

Figure S3. *Cont.*

D) Quantitative RT-PCR experiments on hPSC lines at the pluripotent stage

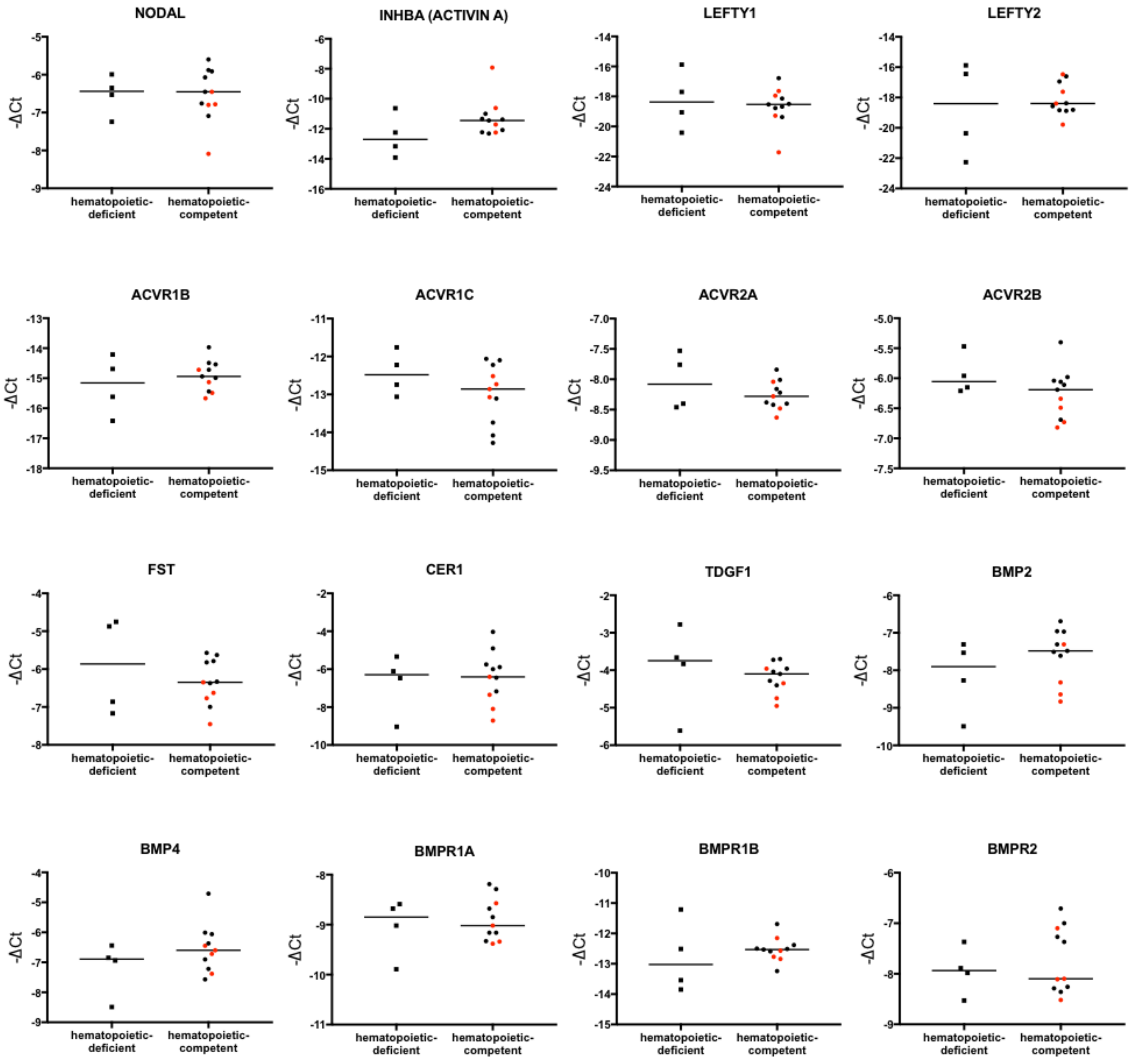

Figure S3. Analysis of the NODAL/ACTIVIN pathway by microarray and qRT-PCR experiments. A) A schematic representation, of the NODAL/ACTIVIN family pathway is shown. B) Several actors of this pathway (in red) were studied at the transcription level by qRT-PCR at the pluripotent stage (1) and/or microarray in hPSCs-derived EBs (2) C) The unsupervised clustering on H1, PB6, PB6.1, PB7 and PB12.1 cell lines (day 16 EBs) did not stratify hPSCs according to their hematopoietic potential (microarray analysis). D) The mRNA expression of several genes from the NODAL/ACTIVIN signaling pathway were also evaluated in the entire panel of 15 hPSC lines, at the pluripotency stage, by qRT-PCR experiments. No significant differential expression was observed between hematopoietic-deficient and -competent hPSCs. Human ES cells are represented in red and hiPSCs in black (*related to Tables S1-S2*).

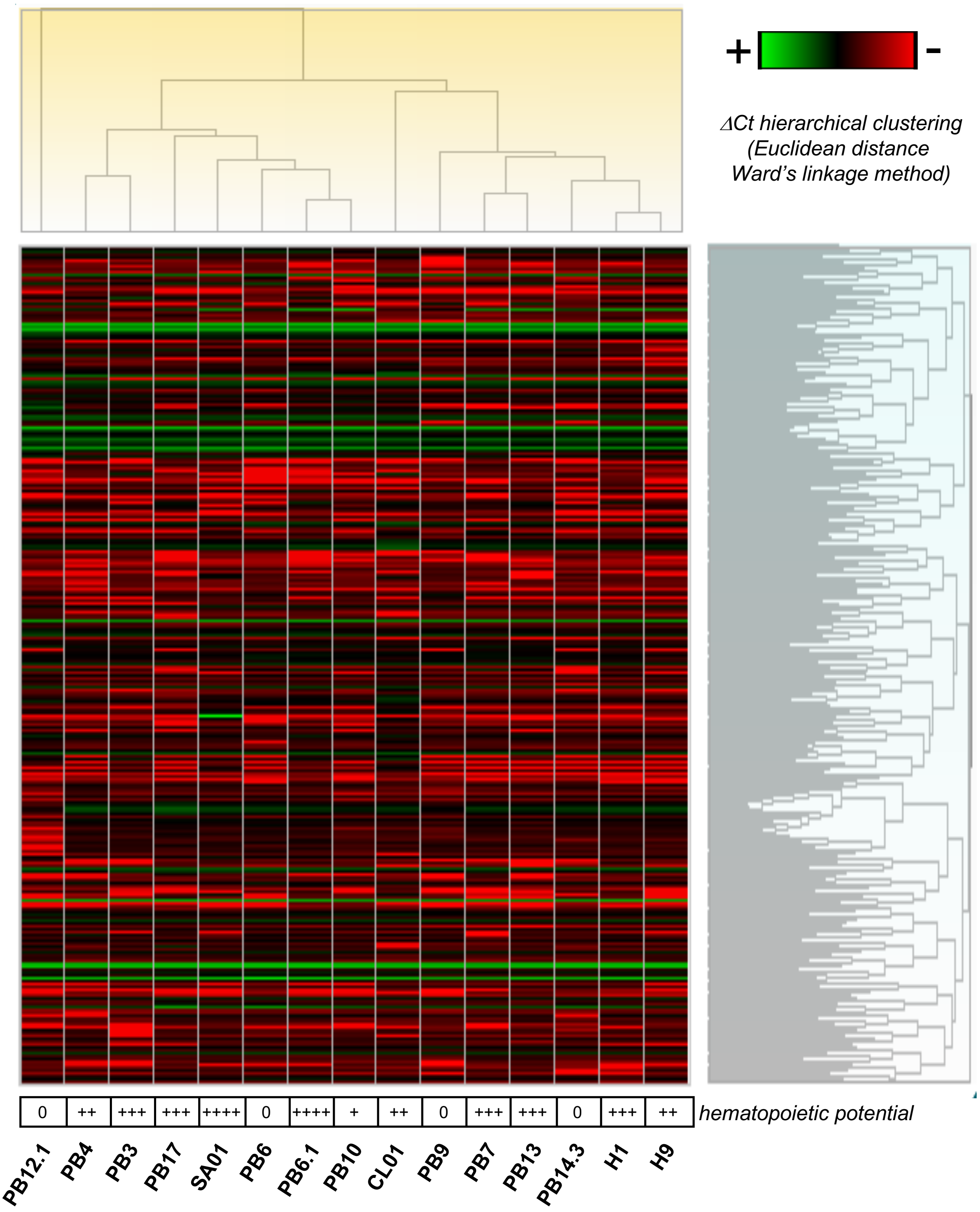

Figure S4. Unsupervised hierarchical clustering of hPSCs at the pluripotent stage according to the expression of 754 miRNAs (TLDA analysis). The unsupervised clustering did not distinguish hematopoietic-competent cells (SA01, CL01, H1, H9, PB3, PB4, PB6.1, PB7, PB10, PB13, PB17) from hematopoietic-deficient ones (PB6, PB9, PB12.1, PB14.3).

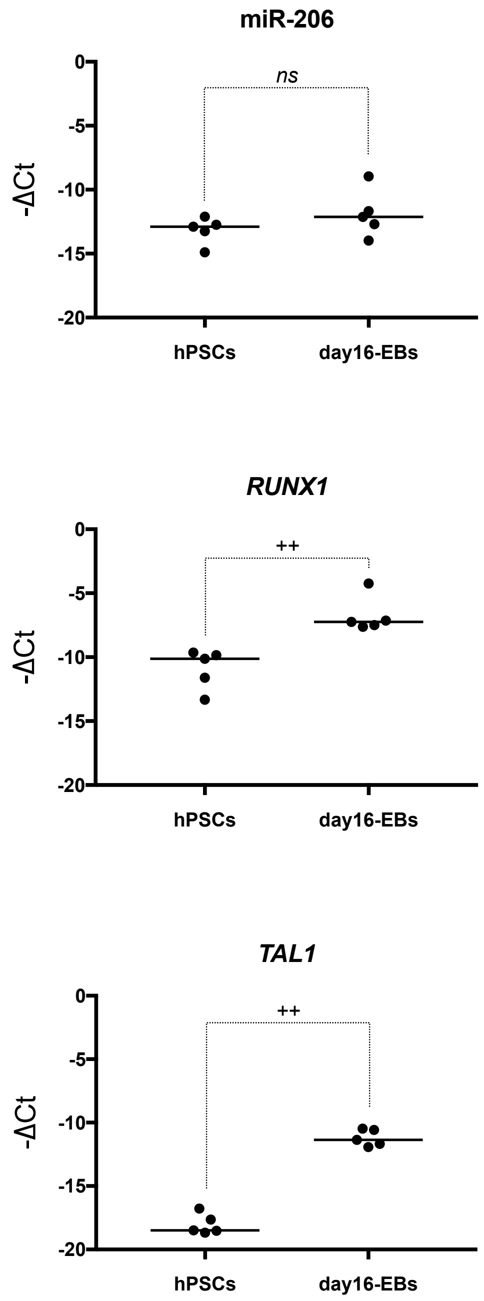

Figure S5. Real-time RT-PCR quantification of miR-206 and *RUNX1*/*TAL1* mRNAs in five hematopoietic-competent hPSCs and day-16 EBs (PB6.1, PB7, SA01, H1, H9). Expression levels are shown as vertical scatter dot plot with median and estimated by a –ΔCt calculation with ΔCt = Ct miRNA – Ct RNU48 (miR-206) or ΔCt = Ct Target – Ct GAPDH (*RUNX1*/*TAL1*). p-values were calculated using the nonparametric 2-tailed Mann-Whitney U-test (ns not significant; + p<0,05; ++ p<0,01; +++ p<0,001).

Table S1. Selected genes analyzed from whole transcriptome data and by qRT-PCR (*related to Figures S1-S3*).

Acronym	Name	Hematopoietic lineage marker gene	Germ layer marker gene			ACTIVIN/NODAL signaling pathway
			ectoderm	endoderm	mesoderm	
ACTC1	actin; alpha; cardiac muscle 1				X	
ACVR1B	activin A receptor; type IB					X
ACVR1C	activin A receptor; type IC					X
ACVR2A	activin A receptor; type IIA					X
ACVR2B	activin A receptor; type IIB					X
AFP	alpha-fetoprotein			X		
AICDA *(AID)*	activation-induced cytidine deaminase	X				
AKT3	v-akt murine thymoma viral oncogene homolog 3	X				
BIC *(MIR155)*	microRNA 155	X				
BMP2	bone morphogenetic protein 2					X
BMP4	bone morphogenetic protein 4					X
BMPR1A	bone morphogenetic protein receptor, type IA					X
BMPR1B	bone morphogenetic protein receptor, type IB					X
BMPR2	bone morphogenetic protein receptor, type II					X
CD34	CD34 molecule	X				
CD48	CD34 molecule	X				
CDKN1B *(P27)*	cyclin-dependent kinase inhibitor 1B	X				
CDKN2A *(P16)*	cyclin-dependent kinase inhibitor 2A	X				
CDKN2B *(P15)*	cyclin-dependent kinase inhibitor 2B	X				
CDX2	caudal type homeobox 2			X		
CER1	cerberus 1 homolog; cysteine knot superfamily (Xenopus laevis)			X		X
COL1A1	collagen; type I; alpha 1				X	
COL2A1	collagen; type II; alpha 1				X	
CXCR4	chemokine (C-X-C motif) receptor 4			X		
DDX4	DEAD (Asp-Glu-Ala-Asp) box polypeptide 4				X	
DKK1	dickkopf homolog 1				X	
E2F2	E2F transcription factor 2	X				
E2F3	E2F transcription factor 3	X				
EPCAM	epithelial cell adhesion molecule			X		
FLT3	fms-related tyrosine kinase 3	X				
FN1	fibronectin 1			X		
FOXA2	forkhead box A2			X		
FOXH1	forkhead box H1				X	X
FST	follistatin					X
GATA1	GATA binding protein 1	X				
GATA4	GATA binding protein 4			X	X	
GATA6	GATA binding protein 6			X	X	
GSC	goosecoid			X		
HAND1	heart and neural crest derivatives expressed 1				X	
HBB	hemoglobin, beta				X	
HNF1B	HNF1 homeobox B			X		
HNRNPA1	heterogeneous nuclear ribonucleoprotein A1	X				

Table S1. *Cont.*

Acronym	Name	Hematopoietic lineage marker gene	Germ layer marker gene			ACTIVIN/NODAL signaling pathway
			ectoderm	endoderm	mesoderm	
HNRNPA2B1 *(HNRNPA2)*	heterogeneous nuclear ribonucleoprotein A2/B1	X				
HNRNPK	heterogeneous nuclear ribonucleoprotein K	X				
HNRPA3	heterogeneous nuclear ribonucleoprotein A3	X				
HOXA9	homeobox A9	X				
INHBA *(ACTIVIN)*	inhibin, beta A (activin A)					X
ISL1	ISL LIM homeobox 1				X	
KDR	kinase insert domain receptor				X	
KMT2A *(MLL)*	lysine (K)-specific methyltransferase 2A	X				
KRT18	keratin 18			X		
LEFTY1	left-right determination factor 1					X
LEFTY2	left-right determination factor 2					X
MEF2C	myocyte enhancer factor 2C				X	
MESP1	mesoderm posterior 1 homolog (mouse)				X	
MIXL1	Mix1 homeobox-like 1	X		X		
MSX1	msh homeobox 1				X	
MTOR	mechanistic target of rapamycin	X				
MYF5	myogenic factor 5				X	
NCAM1	neural cell adhesion molecule 1			X		
NES	nestin		X			
NKX2-5	NK2 Homeobox 5				X	
NODAL	nodal homolog (mouse)					X
NUMB	numb homolog (Drosophila)	X				
PAX6	paired box 6		X			
PCBP2 *(HNRPE2)*	poly(rC) binding protein 2	X				
PIK3CD	phosphatidylinositol-3-kinase; catalytic; delta polypeptide	X				
PIK3CG	phosphatidylinositol-3-kinase; catalytic; gamma polypeptide	X				
RUNX1*(AML1)*	runt-related transcription factor 1	X				
SLAMF1 *(CD150)*	signalling lymphocytic activation molecule family member 1 (CD150)	X				
SOX1	SRY (sex determining region Y)-box 1		X			
SOX17	SRY (sex determining region y)-box 17			X		
SOX2	SRY (sex determining region Y)-box 2		X			
SPI1 *(PU.1)*	spleen focus forming virus (SFFV) proviral integration oncogene spi1	X				
T	brachyury homolog (mouse)	X		X	X	
TAL1 *(SCL)*	T-cell acute lymphocytic leukemia 1	X				
TBX20	T-box 20				X	
TBX5	T-box 5				X	
TDGF1 *(CRIPTO1)*	teratocarcinoma-derived growth factor 1					X
TUBB3	tubulin; beta 3		X			
WNT3A	wingless-type MMTV integration site family, member 3A				X	
WT1	Wilms tumor 1				X	
ZFPM2	zinc finger protein, multitype 2				X	
ZIC1	Zic family member 1 (odd-paired homolog; Drosophila)		X			

Table S2. Primer list for qRT-PCR analysis of the NODAL/ACTIVIN signaling pathway (*related to Figure S3*).

Gene	Accession #	Forward primer (5'-3')	Reverse primer (5'-3')
GAPDH	NM_002046	TGCACCACCAACTGCTTAGC	GGCATGGACTGTGGTCATGAG
ACVR1B (ALK4)	NM_004302	GAGGAAATGCGAAAGGTTGT	AGCTGGGAGAGGGTCTTCTT
ACVR1C (ALK7)	NM_145259	TGCCTTCAGATCCCTCGATA	GCATACCAACACTCACGCATT
ACVR2A	NM_001216	GAGGTACATGGCTCCAGAGG	TTTCCTCCTCAAATGGCAAC
ACVR2B	NM_001106	AAGCCGTCTATTGCCCACAG	GAGCCATGTACCGTCTCGTG
BMP2	NM_001200	CCCAGCGTGAAAAGAGAGAC	GGAAGCAGCAACGCTAGAAG
BMP4	NM_001202	AGCCATTCCGTAGTGCCATC	CGACCATCAGCATTCGGTTA
BMPR1A (ALK3)	NM_004329	CTGGGGTCCGGACTTATGA	TGCTTTCTTACGACTCCTCCA
BMPR1B (ALK6)	NM_001203	TCATCCTTTGGGAGGTTGCT	GGGTCACTGGGCACTAGGTC
BMPR2	NM_001204	AAAGCCCAGAAGAGCACAGA	CTTCACAGTCCAGCGATTCA
CER1 (CERBERUS)	NM_005454	CTTCTCAGGGGGTCATCTTG	TCCCAAAGCAAAGGTTGTTC
CRIPTO (TDGF1)	NM_003212	TAACGCCTCTTTTCCCCCTAAT	TCATCTCTGAAGGCCAGGTATC
FST (FOLLISTATIN)	NM_006350	GGGAATGATGGAGTCACCTACT	CCAACCTTGAAATCCCATAAAC
INHBA (ACTIVIN)	NM_002192	TGAATGAACTTATGGAGCAGACC	GGGACTTTTAGGAAGAGCCAGA
LEFTY1	NM_020997	CCAGTACGTGGCCCTGCT	CTGCTCCATGCCGAACAC
LEFTY2	NM_003240	CGTGAGGGCCCAGTATGTAGT	CTGCTCCATGCCGAACAC
NODAL	NM_018055	GAGGAGTTTCATCCGACCAA	GCACTCTGCCATTATCCACA

Table S3. Hematopoiesis-related miRNAs selected for kinetic expression analysis (*related to Figure 2*).

**miRNA (miRBase ID)**	**expressed in**											
	**PSCs**	**HSCs**	**MYELOID PROGENITORS**	*Megakaryocytes*	*Erythrocytes*	*Macrophages*	*Granulocytes*	**LYMPHOID PROGENITORS**	*T cells*	*B cells*	**HEMATOLOGICAL MALIGNANCIES**	**References**
hsa-miR-125b-5p		**X**	**X**					**X**			**X**	Ooi et al. Proc Natl Acad Sci U S A 2010; Shaham et al. Leukemia 2012
hsa-miR-142-3p		**X**						**X**	X		**X**	Dahlhaus et al. Neoplasma 2013
hsa-miR-150-5p			**X**	X				**X**	X	X	**X**	Garzon et al. Curr Opin Hematol 2008; Vasilatou et al. Eur J Haematol 2010; Yeh et al. Mol Cancer 2016
hsa-miR-155-5p	**X**	**X**	**X**	X	X	X	X	**X**	X	X	**X**	Georgantas et al. Proc Natl Acad Sci U S A 2007; Mayani Stem Cells Dev 2010; Yeh et al. Mol Cancer 2016
hsa-miR-223-3p			**X**				X				**X**	Johnnidis et al. Nature 2008; Fazi et al. Cancer Cell 2007; Yeh et al. Mol Cancer 2016
hsa-miR-302-3p(1)	**X**											Subramanyam et al. Nat Biotechnol 2011; Leonardo et al. Nat Cell Biol 2012

PSCs, pluripotent stem cells; HSCs, hematopoietic stem cells

(1) primer for miR-302 recognizes all four isoforms (see also Table S7).

Table S4. miRNAs differentially expressed between hematopoietic-deficient and hematopoietic-competent hPSCs (TLDA experiments, related to Figure 3A).

miRNA (miRBase ID)	hPSCs tested (ES + iPS or iPS alone)	Fold change in miRNA expression (hematopoietic-competent *vs* -deficient hPSCs)	Statistical significance	
			LiMMa	LiMMa Bonferroni correction
hsa-miR-105-5p	ES + iPS	X 0.066	+	
	iPS alone	X 0.054	+	
hsa-miR-106a-3p	iPS alone	X 9.2	+	
hsa-miR-122-5p	ES + iPS	X 83.3	++	
	iPS alone	X 142.8	++	
hsa-miR-135b-3p	ES + iPS	X 0.4	+	
**hsa-miR-206**	**ES + iPS**	**X 0.043**	**+++**	**++**
	**iPS alone**	**X 0.041**	**+++**	**++**
hsa-miR-296-3p	ES + iPS	X 1.8	++	
	iPS alone	X 1.9	+	
hsa-miR-335-5p	ES + iPS	X 1.9	+	
	iPS alone	X 2.1	+	
hsa-miR-492	ES + iPS	X 0.48	+	
hsa-miR-515-3p (1)	ES + iPS	X 3.5	+	
hsa-miR-520a-3p (1)	ES + iPS	X 13.0	++	
	iPS alone	X14.1	+	
hsa-miR-622	ES + iPS	X 0.11	+	
	iPS alone	X 0.058	+	

(1): member of the C19MC cluster located within chromosome 19.
ES + iPS, analysis of the entire panel of 15 hPSC lines; iPS alone, analysis restricted to hiPSC lines.
+ p<0,05; ++ p<0,01; +++ p<0,001.
LiMMa, Linear models for microarrays.

Table S5. miRNAs differentially expressed between high and poor hematopoietic-competent hPSCs (TLDA experiments, related to Figure 3B).

miRNA (miRBase ID)	Fold change in miRNA expression (High hematopoietic-competent *vs* poor hematopoietic-competent hPSCs)	Statistical significance	
		LiMMa	LiMMa Bonferroni correction
hsa-miR-15b-3p	X 0.48	+	
hsa-miR-22-5p	X 0.42	+	
hsa-miR-34c-5p	X 3.1	++	
hsa-miR-138-5p	X 0.62	+	
hsa-miR-190a-5p	X 4.8	+	
hsa-miR-192-3p	X 25.0	++	
hsa-miR-328-3p	X 0.45	+	
hsa-miR-345-5p	X 0.52	+	
hsa-miR-378a-5p	X 7.6	+	
**hsa-miR-520a-3p (1)**	**X 33.2**	**++**	**+**
hsa-miR-941	X 13.0	+	

(1): member of the C19MC cluster located within chromosome 19.
+ p<0,05; ++ p<0,01; +++ p<0,001.
LiMMa, Linear models for microarrays.

Table S6. Genes potentially up-regulated by a reduced expression of miR-206 in the context of hematopoietic-competent hPSC (*related to Figure 4*).

**Target gene**		**p-values (Up)**	**Fold Change Hematopoietic-competent *vs* -deficient hPSCs**	**Biological process**	
ABCA1	ATP binding cassette subfamily A member 1	0.0044	X 2.48	Transporter activity	Transport
ANXA2	annexin A2	0.0098	X 2.25	Calcium ion binding	Signal transduction ; Cell communication
CA12	carbonic anhydrase 12	0.0033	X 2.65	Catalytic activity	Metabolism ; Energy pathways
CDR1	cerebellar degeneration related protein 1	0.00557	X 2.60	Molecular function unknown	Biological_process unknown
COL19A1	collagen type XIX alpha 1 chain	0.00934	X 1.86	Extracellular matrix structural constituent	Cell growth and/or maintenance
CSF1	colony stimulating factor 1	0.018	X 2.19	Cytokine activity	Cell proliferation ; Cell differentiation ; Signal transduction ; Hemopoiesis
DLG2	discs large MAGUK scaffold protein 2	0.016	X 3.86	Cell adhesion molecule activity	Cell communication ; Signal transduction
ECM2	extracellular matrix protein 2	8.58E-5	X 5.08	Extracellular matrix structural constituent	Cell growth and/or maintenance
EIF4E	eukaryotic translation initiation factor 4E	0.0489	X 1.68	Translation regulator activity	Protein metabolism
ELMO1	engulfment and cell motility 1	0.0146	X 2.01	Motor activity	Cell growth and/or maintenance
ENTPD7	ectonucleoside triphosphate diphosphohydrolase 7	0.0233	X 1.88	Hydrolase activity	Metabolism ; Energy pathways
FAM91A1	family with sequence similarity 91 member A1	0.0468	X 1.65	Molecular function unknown	Biological process unknown
FN1	fibronectin 1	0.0181	X 2.04	Extracellular matrix structural constituent	Cell growth and/or maintenance
FZD4	frizzled class receptor 4	0.0255	X 1.85	G-protein coupled receptor activity	Cell communication ; Signal transduction
GCH1	GTP cyclohydrolase 1	0.011	X 2.17	Hydrolase activity	Metabolism ; Energy pathways
GDF6	growth differentiation factor 6	0.0291	X 1.44	Growth factor activity	Cell communication ; Signal transduction

Table S6. *Cont.*

Target gene		p-values (Up)	Fold Change Hematopoietic-competent *vs* -deficient hPSCs	Biological process	
GLS	glutaminase	0.011	X 2.16	Hydrolase activity	Metabolism ; Energy pathways
GUCY1A2	guanylate cyclase 1 soluble subunit alpha 2	0.00422	X 2.56	Guanylate cyclase activity	Cell communication ; Signal transduction
HNF4A	hepatocyte nuclear factor 4 alpha	8.58E-5	X 8.56	Ligand-dependent nuclear receptor activity	Cell communication ; Signal transduction
HNRNPU	heterogeneous nuclear ribonucleoprotein U	0.0438	X 1.68	RNA binding	Regulation of nucleobase, nucleoside, nucleotide and nucleic acid metabolism
KANK4	KN motif and ankyrin repeat domains 4	0.009	X 2.27	Molecular function unknown	Biological process unknown
KCNJ15	potassium voltage-gated channel subfamily J member 15	0.0163	X 1.80	Inward rectifier channel	Transport
KDELR2	KDEL endoplasmic reticulum protein retention receptor 2	0.0219	X 1.91	Transporter activity	Transport
KIAA0040	KIAA0040	4.77E-4	X 3.56	Molecular function unknown	Biological process unknown
JCAD (KIAA1462)	junctional cadherin 5 associated	0.009	X 2.31	Molecular function unknown	Biological process unknown
PLPPR4 (LPPR4)	phospholipid phosphatase related 4	0.005	X 2.47	Lipid phosphatase activity	Cell communication ; Signal transduction
MAPKBP1	mitogen-activated protein kinase binding protein 1	0.0234	X 1.86	Molecular function unknown	Biological process unknown
METTL7A	methyltransferase like 7A	2.86E-4	X 3.90	Methyltransferase activity	Metabolism; Energy pathways
MPP7	membrane palmitoylated protein 7	0.0298	X 1.82	Receptor signaling complex scaffold activity	Cell communication ; Signal transduction
MR1	major histocompatibility complex, class I-related	0.0032	X 2.62	MHC class I receptor activity	Immune response
MYOCD	myocardin	8.58E-5	X 7.36	Transcription factor activity	Regulation of nucleobase, nucleoside, nucleotide and nucleic acid metabolism
NAALADL1	N-acetylated alpha-linked acidic dipeptidase like 1	0.0117	X 2.16	Dipeptidase activity	Biological_process unknown

Table S6. *Cont.*

Target gene		p-values (Up)	Fold Change Hematopoietic-competent *vs* -deficient hPSCs	Biological process	
NR1H3	nuclear receptor subfamily 1 group H member 3	0.0281	X 1.82	Ligand-dependent nuclear receptor activity	Cell communication ; Signal transduction
OSTF1	osteoclast stimulating factor 1	0.00357	X 2.56	Receptor signaling complex scaffold activity	Cell communication ; Signal transduction
PAX6	paired box 6	0.0478	X 1.57	Transcription factor activity	Regulation of nucleobase, nucleoside, nucleotide and nucleic acid metabolism
PDGFA	platelet derived growth factor subunit A	0.00381	X 2.65	Growth factor activity	Cell proliferation ; Cell surface receptor linked signal transduction ; Cell migration
RABGAP1L	RAB GTPase activating protein 1 like	0.0075	X 2.23	GTPase activator activity	Biological process unknown
RUNX1	runt related transcription factor 1	0.0001	X 4.5	Transcription factor activity	Regulation of nucleobase, nucleoside, nucleotide and nucleic acid metabolism
SCD	stearoyl-CoA desaturase	0.0428	X 1.69	Oxidoreductase activity	Metabolism ; Energy pathways
SLC15A2	solute carrier family 15 member 2	0.00435	X 2.59	Auxiliary transport protein activity	Transport
SLC16A3	solute carrier family 16 member 3	0.00563	X 2.52	Auxiliary transport protein activity	Transport
SLC25A30	solute carrier family 25 member 30	0.0175	X 1.93	Transporter activity	Transport
SLC31A1	solute carrier family 31 member 1	0.0419	X 1.75	Transporter activity	Transport
SLC35B3	solute carrier family 35 member B3	0.0415	X 1.72	Auxiliary transport protein activity	Transport
SLC44A1	solute carrier family 44 member 1	0.034	X 1.74	Molecular function unknown	Transport
SLC45A4	solute carrier family 45 member 4	0.0452	X 1.71	Molecular function unknown	Transport
SLC6A13	solute carrier family 6 member 13	0.0183	X 2.01	Auxiliary transport protein activity	Transport
SLC7A11	solute carrier family 7 member 11	0.0263	X 1.82	Auxiliary transport protein activity	Transport

Table S6. *Cont.*

Target gene		p-values (Up)	Fold Change Hematopoietic-competent *vs* -deficient hPSCs	Biological process	
SLC7A2	solute carrier family 7 member 2	2.67E-4	X 4.15	Auxiliary transport protein activity	Transport
SPATS2L	spermatogenesis associated serine rich 2 like	0.037	X 1.74	Molecular function unknown	Biological process unknown
SPTLC3	serine palmitoyltransferase long chain base subunit 3	0.00143	X 3.25	Transferase activity	Biological process unknown
STC2	stanniocalcin 2	0.04	X 1.77	Hormone activity	Cell communication ; Signal transduction
TAL1	TAL bHLH transcription factor 1, erythroid differentiation factor	9.54E-6	X 6.29	Transcription factor activity	Regulation of nucleobase, nucleoside, nucleotide and nucleic acid metabolism
TBX18	T-box 18	0.00334	X 2.75	Transcription factor activity	Transcription
TCF7L2	transcription factor 7 like 2	0.0427	X 1.73	Transcription factor activity	Regulation of nucleobase, nucleoside, nucleotide and nucleic acid metabolism
THRB	thyroid hormone receptor beta	0.0299	X 1.82	Ligand-dependent nuclear receptor activity	Cell communication ; Signal transduction
TMEM120B	transmembrane protein 120B	0.0426	X 1.71	Molecular function unknown	Biological process unknown
TPK1	thiamin pyrophosphokinase 1	9.54E-6	X 6.16	Catalytic activity	Metabolism ; Energy pathways
TPM3	tropomyosin 3	0.0316	X 1.79	Cytoskeletal protein binding	Cell growth and/or maintenance
TSPAN9	tetraspanin 9	0.0214	X 1.91	Cell adhesion molecule activity	Cell communication ; Signal transduction
UHRF1BP1L	UHRF1 binding protein 1 like	0.0219	X 1.92	Molecular function unknown	Biological process unknown
ZC3HAV1	zinc finger CCCH-type containing, antiviral 1	0.004	X 3.18	Defense/immunity protein activity	Immune response

Table S7. Primer list for qRT-PCR analysis of hematopoiesis-related miRNAs (*related to Figure 2*).

miRNA name	primer sequence (5'-3')
**hsa-miR-125b-5p**	TTGTTTCCCTGAGACCCTAACTTG
**hsa-miR-142-3p**	GGGTGGTTGTAGTGTTTCCTACT
**hsa-miR-150-5p**	AACCTGATCTCCCAACCCTTGTA
**hsa-miR-155-5p**	GGGTCTTAATGCTAATCGTGATAGG
**hsa-miR-223-3p**	CCACGTCTGTCAGTTTGTCAAA
**hsa-miR-302-3p**(1)	GGTTAAGTGCTTCCATGTTT

(1) primer for miR-302 recognizes all four isoforms (a/b/c/d)
